# Midterm review of national health plans: an example from the United Republic of Tanzania

**DOI:** 10.2471/BLT.14.141069

**Published:** 2015-02-19

**Authors:** Leonard EG Mboera, Yahya Ipuge, Claud J Kumalija, Josbert Rubona, Sriyant Perera, Honorati Masanja, Ties Boerma

**Affiliations:** a National Institute for Medical Research, PO Box 9653, Dar es Salaam, United Republic of Tanzania.; b Ifakara Health Institute, Dar es Salaam, United Republic of Tanzania.; c Ministry of Health and Social Welfare, Dar es Salaam, United Republic of Tanzania.; d CTS Global USA/Center for Disease Control and Prevention, Dar es Salaam, United Republic of Tanzania.; e World Health Organization, Geneva, Switzerland.

## Abstract

In the health sector, planning and resource allocation at country level are mainly guided by national plans. For each such plan, a midterm review of progress is important for policy-makers since the review can inform the second half of the plan’s implementation and provide a situation analysis on which the subsequent plan can be based. The review should include a comprehensive analysis using recent data – from surveys, facility and administrative databases – and global health estimates. Any midterm analysis of progress is best conducted by a team comprising representatives of government agencies, independent national institutions and global health organizations. Here we present an example of such a review, done in 2013 in the United Republic of Tanzania. Compared to similar countries, the results of this midterm review showed good progress in all health indicators except skilled birth attendance.

## Résumé

Dans le secteur de la santé, la planification et l'allocation des ressources au niveau national sont principalement guidées par les plans des pays. Pour chacun de ces plans, un examen à mi-parcours des progrès réalisés est important pour les responsables politiques, puisque cet examen peut donner des informations pour la deuxième moitié de la mise en œuvre du plan et fournir une analyse de la situation à partir de laquelle la suite du plan peut se baser. L'examen devrait inclure une analyse complète utilisant les données récentes (obtenues à partir des bases de données des enquêtes, des établissements et de l'administration) et les estimations sanitaires mondiales. Toute analyse à mi-parcours des progrès réalisés doit être menée de préférence par une équipe comprenant des représentants des agences gouvernementales, des institutions nationales indépendantes et des organisations sanitaires mondiales. Nous présentons ici un exemple d'un tel examen, réalisé en 2012 dans la République-Unie de Tanzanie. Par rapport à d'autres pays similaires, les résultats de cet examen à mi-parcours montrent une progression satisfaisante de tous les indicateurs de santé, à l'exception de l'accouchement assisté par un soignant qualifié.

## Resumen

En el sector de la salud, la planificación y la asignación de recursos a nivel nacional se guían principalmente por planes nacionales. Para los planificadores de políticas es importante contar una revisión intermedia de los progresos de cada plan, ya que puede proporcionar información sobre la segunda mitad de la implementación del plan y un análisis de la situación en la que basar planes posteriores. La revisión debe incluir un análisis exhaustivo con datos recientes - a partir de encuestas y bases de datos administrativas y de los centros - y estimaciones mundiales de salud. La mejor manera de realizar cualquier análisis intermedio de los progresos es a través de un equipo integrado por representantes de organismos gubernamentales, instituciones nacionales independientes y organizaciones mundiales de salud. Aquí presentamos un ejemplo de ese tipo de revisiones, realizada en 2012 en Tanzania. En comparación con países similares, los resultados de este examen intermedio mostraron un progreso correcto en todos los indicadores de salud, excepto en la prestación de atención especializada en el parto.

## ملخص

يتم الاسترشاد بصورة رئيسية بالخطط الوطنية في تخطيط وتخصيص الموارد على صعيد البلدان في قطاع الصحة. ويعد استعراض منتصف المدة للتقدم المحرز مهماً لواضعي السياسات بالنسبة لكل خطة من هذه الخطط حيث يمكن الاستنارة بالاستعراض في النصف الثاني من تنفيذ الخطة ويمكنه تقديم تحليل للوضع بشأن ما يمكن أن تستند عليه الخطة اللاحقة. وينبغي أن يتضمن الاستعراض تحليلاً شاملاً باستخدام البيانات الحديثة - المستمدة من المسوح والمرافق وقواعد البيانات الإدارية - وتقديرات الصحة على الصعيد العالمي. ومن الأفضل إجراء أي تحليل في منتصف المدة للتقدم المحرز بواسطة فريق يتألف من ممثلي الوكالات الحكومية والمؤسسات الوطنية المستقلة ومنظمات الصحة على الصعيد العالمي. ونقدم هنا مثالاً على هذا الاستعراض، الذي تم إجراؤه في عام **2012 ** في جمهورية تنزانيا المتحدة. ومقارنة ببلدان مشابهة، أظهرت نتائج استعراض منتصف المدة تقدماً جيداً في جميع مؤشرات الصحة باستثناء خدمات التوليد التي يقدمها أشخاص مهرة.

## 摘要

在卫生部门，国家层级的规划和资源配置主要由国家计划指导。对于每一个这样的计划，期中进展评审对决策者来说非常重要，因为评审可以为计划后半段的实施提供信息，并提供形势分析作为后续计划的基础。评审应包括使用最近的数据（来自调查、设施和管理数据库）和全球健康估计的综合分析。任何期中进展分析最好由政府机构、独立的国家机构和全球卫生组织的代表组成的团队执行。这里我们介绍的评审例子是2012年在坦桑尼亚完成的。与相似的国家相比，这次期中评审的结果显示除熟练助产服务之外的所有指标都进展良好。

## Резюме

В секторе здравоохранения планирование и распределение ресурсов на уровне страны определяется, главным образом, национальными планами. По каждому подобному плану директивным органам важно проводить промежуточный обзор хода его реализации, поскольку данный обзор может повлиять на выполнение второй половины плана и представить данные для ситуационного анализа, на котором будет основываться последующий план. Обзор должен включать всесторонний анализ с использованием недавно полученных данных – из баз данных медицинских и административных учреждений, а также проведенных исследований – и глобальных оценок состояния здоровья. Любой промежуточный анализ хода выполнения плана наилучшим образом проводится командой, состоящей из представителей государственных органов, независимых национальных институтов и всемирных организаций здравоохранения. В настоящей статье представлен пример подобного обзора, выполненного в 2012 году в Объединенной Республике Танзания. По сравнению с аналогичными странами результаты этого промежуточного обзора продемонстрировали хорошие результаты по всем показателям здоровья, за исключением квалифицированного родовспоможения.

## Introduction

In the health sector, regular reviews of progress and performance are critical for good planning and resource allocation. Most countries have a national strategic plan for the health sector that outlines major strategies and sets targets. Such plans often cover a period of five years and usually include regular monitoring of core indicators and the progress being made towards set targets. The results of progress reviews are typically published in annual progress reports^1^^–^^5^ and discussed at annual meetings of the relevant stakeholders. The stakeholders may then decide how – and if – the implementation of the strategic plan needs to be adjusted. In a recent investigation of 75 countries, it was found that 58 (77%) countries were conducting annual reviews of their health sectors.^6^ During the implementation of a strategic plan, such annual reviews are often supplemented with more extensive midterm and – sometimes – final reviews. The World Health Organization (WHO) has specified the key characteristics of a monitoring and evaluation platform for supporting regular reviews of health sector strategy.^7^

Compared with annual reviews, midterm reviews are usually broader in process and scope. They may be conducted in conjunction with a regular annual review or they may replace it.^6^^,^^7^ They take a multi-year perspective and pull together all of the available relevant data to assess the progress and performance of the health sector as a whole. Ideally, they should pay special attention to data quality, long-term trends, contextual changes and equity, and compare performance at national level with that in other similar countries. They should also form the basis for the situation analysis for the next strategic plan.

At present, most midterm reviews are conducted by teams of international and local consultants and the analysis and synthesis of evidence are usually quite rapid.^6^ Ideally, any midterm review should begin with a thorough and systematic analysis and synthesis of all of the relevant data, to provide a comprehensive picture of progress and performance. It should be conducted by national research and public health institutions, in close collaboration with the national health ministry, the national statistical authority and international partners.

As an example, we present a midterm review done for the United Republic of Tanzania in 2013. A systematic approach was used to analyse and synthesize data from multiple sources.

## Context of the review

The Ministry of Health and Social Welfare of the United Republic of Tanzania is currently implementing its third national plan, which covers the period 2009–2015.^8^ This plan represents the key government document that provides specific guidance to the health sector. The main aim of the plan is improving access to those health services that are relevant to the Millennium Development Goals – at community, facility and district levels. The plan pays special attention to equity and gender issues and the general improvement of service quality. There are baseline values available – for 2008–2009 or earlier – for most of the 42 indicators used to track progress with the plan’s implementation and corresponding target values for 2015.

The results of household surveys and longitudinal community studies have indicated that the United Republic of Tanzania has made major progress towards the achievement of its health goals since the mid-1990s.^9^ In particular, the child mortality rate declined substantially between 1995 and 2005. Since 1999, health sector reviews have been conducted on an annual basis and a health sector performance profile has been produced.^3^ Our review – done in the context of general midterm policy review – included several sessions to report midterm progress to the Ministry of Health and Social Welfare and relevant development partners.

## Methods

The review team organized the work into four main elements (Box 1). To assess progress systematically, the national plan’s indicators: six input, five output and 14 intervention coverage – including reductions in risk behaviours – and 17 impact or health status measures (Table 1) were put together in a stepwise logical results framework.^7^ This framework was designed to provide a general overview of where investments had been successful and whether more investments were likely to lead to better results.

Box 1 **Key components of an analytical midterm review of the United Republic of Tanzania’s strategic plan for health in 2009–2015**

**Core indicators and targets**

- Map the core indicators of the health sector’s strategic plan on to the logical results framework.
- Update baseline data where new data are available.

**Data sources**

- Use all relevant data, including routine health facility data and those from household, special population and facility surveys and administrative data sources.
- Assess data quality by considering trends over time and internal consistency of levels and trends at regional – or other, subnational – levels and by comparing results from surveys with facility-based data.
- Make transparent adjustments, where needed, to correct improbable values for indicators that affect the overall analysis.
- Archive and make the dataset used for the synthesis available.

**Analysis and synthesis**

- Wherever the relevant data are available, assess progress in terms of each of the core indicators, focusing on the first half of the period covering the plan’s implementation.
- Where relevant and possible, assess inequalities in demographic, socioeconomic and geographical stratifiers.
- Compare national progress and performance on selected indicators with those of countries that have comparable data and estimates.

**Communication of results**

- Create (i) a comprehensive report organized by the key areas of the strategic plan, (ii) a set of statistical profiles for all major subnational areas, and (iii) a policy brief that summarizes the main results.
- Present the results to – and discuss them with – the midterm review team and Ministry of Health
- Present the main findings at the plan’s next annual review.

**Table 1:** Midterm progress in the health sector’s national strategic plan for 2009–2015, United Republic of Tanzania

Health indicator	Data source	Baseline			Achievement		Target value^a^	Inequity
		Year	Value		Year	Value		
Life expectancy for males/females, years	PS/C	2002	51/52		2011	58/61	59/62	ND
Child mortality rate, deaths per 1000 live births	PS/C	2004–2008	91		2006–2010	81	54	ND
Neonatal mortality rate, deaths per 1000 live births	PS/C	2004–2008	29		2006–2011	26	19	ND
Infant mortality rate, deaths per 1000 live births	PS/C	2004–2008	58		2006–2012	51	NS	ND
Stunting among children younger than five years, %	PS/C	2004–2005	38		2010	35	27	S, R and W
Underweight among children younger than five years, %	PS/C	2004–2005	22		2010	14	14	ND
Maternal mortality ratio, deaths per 100 000 live births	PS/C	1996–2005	578		2004–2010	454	156	ND
Total fertility rate, live births per woman	PS/C	2003–2005	5.7		2008–2010	5.4	5.1	R and W
Adolescent fertility rate, % of women who either had a live birth or were pregnant with first child, before 20 years of age	PS/C	2003–2005	52		2010	44	39	R and W
HIV prevalence, %								
Females/males aged 15–24 years	PS/C	2008	3/1.1		2011–2012	2.8/1.2	NS	S
Pregnant women	HFD	2005–2006	6.8		NA	NA	NS	ND
People aged15–49 years	PS/C	2007–2008	5.8		2011–2012	5.3	NS	ND
Tuberculosis notification rate, cases per 100 000 population	HFD	2008	159		2011	142	NS	S
Cholera incidence	HFD	2008	2391		2011	343	0	ND
Cholera case fatality, %	HFD	2008	3		2011	4 (98/2391)	< 1	ND
Malaria prevalence among outpatients younger than five years, %	HFD	2009	40		2012	33	NS	ND
Prevalence of malarial parasitaemia among children younger than five years, %	PS/C	2008	18		2012	9.2	5	ND
Coverage, % of target group								
Measles immunization (0–11 months)	HFD	2008	91		2012	100	85	ND
Third dose of diphtheria–tetanus–pertussis*–Hib (0–11 months)*	HFD	2008	92		2012	95	85	ND
Two-dose vitamin A (6–59 months)	PS/C	2004–2005	46		2010	60	90	S and W
Second dose of tetanus toxoid during pregnancy	PS/C	2000–2005	5		2011	88	90	ND
First antenatal care visit at more than 16 weeks’ gestation, % of pregnant women	PS/C	2000–2005	14		2006–2010	15	60	R
At least four antenatal care visits, % of pregnant women	PS/C	2000–2005	64		2009–2010	36	90	R
Births in health facilities, %	HFD	2007	51		2011	58	70	R and W
Skilled birth attendance, % of births	PS/C	2000–2005	46		2010–2011	56	80	R and W
Contraceptive use, % of married women	PS/C	2004–2005	20		2010	27	60	R and W
Use of insecticide-treated bednets by children/pregnant women, %	PS/C	2008	26/27		2011–2012	73/75	80	ND
Two-dose intermittent preventive treatment against malaria, % of pregnant women	PS/C	2008	30		2011–2012	31	80	ND
Prophylaxis for mother-to-child transmission of HIV, % coverage among pregnant women	HFD	NA	NA		2011	71	80	ND
Antiretroviral coverage, %	HFD	NA	NA		2012	65	60	ND
Tuberculosis treatment success rate, %	HFD	2008	89		2011	88	85	ND
Government expenditure on health, % of total expenditure	AD	2008–2009	12		2012	10	15	ND
Total health expenditure per capita, US$	AD	2008	26		2011	37	NS	ND
Insurance coverage, %	AD	2007	9		2012	14	80	ND
Health workforce, no. per 10 000 population								
Doctors and assistant medical officers	AD	2004–2005	0.7		2012	0.9	NS	ND
Nurses and midwives	AD	2004–2005	2.6		2012	4.9	NS	ND
Pharmacists	AD	2004–2005	0.15		2012	0.12	NS	ND
Outpatient visits per person-year	AD	2012	0.78		2012	0.69	NS	ND
Training institutes with full accreditation	AD	2008	1		2012	56	30	ND
Basic emergency obstetric care, % of facilities	HFD	NA	NA		2012	20	70	ND
Districts with timely surveillance reports, %	AD	2008	60		2012	73	> 90	ND
No. of stockouts of tracer medicines and vaccines	HFD	2006	0		2013	19	NS	ND

AD: administrative data; HFD: health facility data; Hib: *Haemophilus influenzae* type b; HIV: human immunodeficiency virus; NA: not available; ND: none detected; NS: none set; PS/C: population survey or census; R: region of residence; S: sex; US$: United States Dollars; W: wealth quintile.

^a^ To be achieved by the end of 2015.

We used a mix of health facility, administrative and survey data and the results of relevant research studies (Box 2) to estimate progress made since the beginning of the strategic plan.

Box 2 **Data sources used in the analytical midterm review of the United Republic of Tanzania’s strategic plan for health in 2009–2015**

**Population health surveys**

- United Republic of Tanzania Demographic and Health Surveys for 1991, 1996, 1999, 2004–2005 and 2011–2012
- United Republic of Tanzania HIV and Malaria Indicator Surveys for 2003–2004, 2007–2008 and 2011–2012
- National tuberculosis prevalence survey for 2012
- National panel surveys for 2008–2009 and 2010–2011
- Post-campaign immunization coverage survey for 2011

**Health facility data and reports**

- Core Health Management Information System database
- Annual Health Statistics Reports for 2007, 2008, 2009, 2010, 2011 and 2012
- Annual health sector performance profiles for 2007, 2008, 2009, 2010 and 2011–2012
- Programme databases and annual reports of disease-specific programmes
- Facility assessments
- Service Availability and Readiness Assessment (SARA) for 2008–2009: census of all 1297 facilities in national sample of 15 districts^9^
- Service Availability and Readiness Assessment (SARA) for 2012: census of all 656 facilities in national sample of 23 districts^8^

**Administrative data**

- Financing data from annual public expenditure review for 2011–2012 and national health accounts for 2001, 2006 and 2011
- Human resources data from national database within the Human Resources Information System and professional and training institutions database
- Infrastructure data from national database of health facilities within the Human Resources Information System

**Other data**

- Health and Demographic Surveillance Systems for Ifakara, Kisesa and Rufiji districts
- Sentinel panel of districts investigated as part of the sample vital registration with verbal autopsy (SAVVY) scheme – also providing facility-based information

The United Republic of Tanzania has had frequent population-based demographic and health surveys^10^ as well as topic-specific surveys that covered human immunodeficiency virus (HIV), tuberculosis, malaria or other health issues.^11^^,^^12^ We analysed data collected in recent surveys of these types, two nationwide socioeconomic panel surveys^13^ and two health facility surveys.^14^

Health surveys were conducted in 2008–2009^15^ and 2012,^16^ to assess the services available and the readiness of those services in terms of staffing, equipment, medicines and diagnostics. The readiness score was computed for different intervention areas, such as child health or malaria, by averaging the availability of a set of essential items.

The United Republic of Tanzania has a national system for the routine collection of data from health facilities and this system provided regional data on many of the indicators of interest for the period 2009–2012. Although we also had access to crude data in some very recent health facility reports, we were cautious in using them because of the potentially low quality of the numerators – the number of individuals covered by an intervention – and denominators – the estimated size of the population in need of that intervention – that had been used. Wherever possible, we assessed the reliability of these reports against the results of relevant surveys.

For our analysis, we used data from the 2012 national census to estimate denominators.^17^ Coverages for antiretroviral therapy (ART) and prevention of mother-to-child transmission of HIV were estimated using the methods and data of the national acquired immunodeficiency syndrome (AIDS) control programme, the 2012 national census and the 2011 HIV/AIDS indicator survey.^11^ Data on tuberculosis indicators were obtained from the national tuberculosis and leprosy control programme and a recent national survey.^12^

As the results from the 2013 national health accounts exercise were not yet available at the time of our analysis, we used the corresponding WHO estimates.^18^ Health workforce data were obtained from the human resources for health information system of the Ministry of Health and Social Welfare.

The strategic plan has no explicit targets for equity. However, wherever possible – and mainly using household survey data^10^ – we investigated trends in inequalities by sex, age group, wealth quintile, region of residence and urban/rural setting.

We compared the time trends in selected indicators and in health sector efficiency – assessed by comparing selected inputs and results – that we recorded for the mainland health sector of the United Republic of Tanzania with the corresponding values for the following countries; Burundi, the Democratic Republic of the Congo, Ethiopia, Kenya, Malawi, Mozambique, Rwanda, Uganda, Zambia and Zimbabwe. We also made between-country comparisons of mortality and health financing indicators using global estimates produced by WHO and other United Nations agencies. We used data from health surveys that had been implemented between 2009 and 2012 to compare progress on coverage indicators between these countries.

The preparatory stage of the midterm analysis took several months and included meetings to ensure broad participation. The actual analytical work was done over a period of four months, with different partners focusing on specific topics and data sources. The analytical team consisted of representatives of the Ministry of Health and Social Welfare, the National Institute for Medical Research, the Ifakara Health Institute and WHO.

All analyses were conducted in Excel (Microsoft, Redmond, United States of America) or Stata version 12 (StataCorp. LP, College Station, USA). Results are reported as percentages since most of the retrieved data are weighted or derived from multiple computations. Most statistical comparisons were made using *χ^2^* tests.

## Results

A detailed report of the midterm analytical review was published,^19^ and we summarize progress made from 2009 to 2012 and compare this to the 2015 targets in Table 1.

### Progress in core indicators

#### Health of children

According to United Nations projections,^20^ the target child mortality set in the national strategic plan had already been reached by 2012. Levels of child mortality showed reduction of the gaps between urban and rural children and between children in the poorest and those in the wealthiest families. Children receiving pentavalent and measles vaccines and nutritional status had also already reached the targets set in the national plan.

#### Maternal health

The maternal mortality ratio declined slowly, from 578 deaths per 100 000 live births in 1996–2005 to 454 deaths per 100 000 live births in 2004–2010. The target set for 2015 is 156 deaths per 100 000 live births. The percentage of deliveries that occurred in health facilities in the presence of skilled birth attendants had not increased from 2009–2012 and remained well below the 80% target value set for 2015. According to the demographic and health surveys for 2004–2005^21^ and 2006–2010,^10^ there was at least one skilled birth attendant present at 46% and 51% of deliveries, respectively. Panel surveys in 2007–2008 and 2010–2011,^13^ which were based on relatively small samples, revealed slightly higher corresponding values – of 59% and 62%, respectively, for the 2 years preceding each survey. The facility surveys indicated that only modest progress had been made by 2012 in the availability of basic obstetric services.

#### Infectious diseases

Malaria mortality and morbidity declined during the first half of the strategic plan’s implementation, as shown by declining numbers of malaria-related hospital deaths, admissions and outpatient visits and the declining prevalence of parasitaemia recorded in household surveys. The use of insecticide-treated bednets showed a threefold increase between 2007–2008 and 2011–2012 and was already close to the 2015 target by 2012. According to the facility surveys in both 2008–2009 and 2012, 80% of health facilities had artemisinin combination therapy available.

Although HIV transmission appeared to decrease gradually during the first half of the plan’s implementation, the number of people living with HIV remained the same – due to increases in both the population and in survival following treatment. By 2012, the percentage of HIV-positive pregnant women receiving antiretroviral prophylaxis was 71%, 9 percentage points from the 2015 target. The percentage of HIV-positive adults receiving ART in 2012 was already higher than the 60% target for those in need of such treatment, while 48% children in need of ART received the treatment.

In almost every region, case notification rates for tuberculosis decreased between 2008 and 2011. However, the tuberculosis survey of 2012 revealed a higher overall prevalence – 295 cases per 100 000 population. This survey also showed that the frequency of treatment success for tuberculosis was high and already above the 2015 target value.^12^

#### Health services

Rates of outpatient department utilization, which are considered as indicators of general access to health services showed no increase between 2009 and 2012. The facility assessments revealed improvements in the proportion of facilities offering integrated child health services, family planning, ART and malaria treatment – but a decline in care for women giving birth over this period (Table 2). There was a slight improvement in general service readiness – as measured by the availability in health facilities of tracer indicators such as diagnostics and medicines – but the percentage of facilities stocking the drugs used in the first-line treatments of HIV infection and tuberculosis fell.

**Table 2:** Availability and readiness of services in health facilities, United Republic of Tanzania, 2008–2009 and 2012

Type of service	No. of facilities offering service			Mean readiness score		Tracer items used to evaluate readiness
	2008–2009 (*n* = 635)	2012 (*n* = 1297)		2008–2009	2012	
Child health	68	82**		65	69	Trained staff, guidelines, child scale, stethoscope, thermometer, haemoglobin test, oral rehydration salts, cotrimoxazole suspension, paracetamol suspension, vitamin A capsules
Family planning	77	83**		72	72	Trained staff and guidelines, blood pressure monitor, at least two types of contraceptive
Antenatal care	82	85		55	52	Blood pressure monitor, haemoglobin test, urine dipstick for glucose
Childbirth	71	64**		59	63	Intravenous kit, oxytocin, magnesium sulfate
Malaria	97	86**		64	70**	Trained staff, guidelines, diagnostic test, artemisinin combination therapy, sulfadoxine–pyrimethamine
Prevention of mother-to-child transmission of HIV	36	78**		69	69	Guidelines, HIV diagnosis, maternal prophylaxis
Antiretroviral therapy	16	28**		50	36**	First-line antiretroviral drug in stock
Tuberculosis	39	38		82	60**	First-line antituberculosis drugs in stock

HIV: human immunodeficiency virus; **P < 0.01.

### Inequalities

The data from household surveys indicated that, in the first half of the plan’s implementation, there were reductions in inequalities – by sex, urban/rural residence and socioeconomic position – in several indicators, including child mortality and immunization and malaria intervention coverages. For several anthropometric indicators in children and skilled birth attendance, however, large inequalities persisted (Table 1).

Combined analysis of the health facility and survey data indicated that, in general, the health services in western regions were relatively weak whereas those in eastern and northern regions were relatively strong. Further analysis of the regional data showed that some regions performed markedly better than might have been predicted from their level of socioeconomic development.^19^

### Between-country comparisons

In 2012, among the 11 countries we included in our comparison, only Kenya and Zambia had higher gross domestic products per capita than the United Republic of Tanzania and only Rwanda and Uganda had higher total health expenditures per capita than the United Republic of Tanzania.

Although all 11 countries had improved their health services in recent years, the United Republic of Tanzania ranked in the top half – and often in the top three countries – in 2011–2012 for most of the health indicators we investigated (Table 3). For most indicators, the United Republic of Tanzania’s ranking in 2011–2012 was similar to that recorded 5 years earlier. However, the country performed less well in skilled birth attendance than most of the other countries in our comparison – especially in terms of the progress made in such attendance between 2005 and 2011.

**Table 3:** Health indicator rankings before and during the implementation of the United Republic of Tanzania’s national plan for 2009–2015

Health indicator	Before implementation of plan					Middle of plan’s implementation			
	Year	Value^a^	Median for 10 countries^b^	United Republic of Tanzania’s rank^c^		Year	Value^a^	Median for 10 countries^b^	United Republic of Tanzania’s rank^c^
Gross national income per capita, PPP international dollars	2008	1370	880	3		2012	1650	1240	3
Total health expenditure per capita, PPP international dollars	2008	68	65	5		2011	107	77	3
Health workforce, health professionals per 10 000 population	NA	NA	NA	NA		2012	5.9	7.5	5^d^
Mortality rate among children younger than five years, deaths per 1000 live births	2005	90	114	1		2012	55	83	1
Maternal mortality ratio, maternal deaths per 100 000 live births	2005	650	698	4		2012	454	523	2
Stunting among children younger than five years, %	2005	38	47	4		2011	42	43	5
Family planning, % of married women with demand satisfied	2005	52	52	5		2011	61	64	6
Skilled birth attendance, % of births	2005	44	43	5		2011	49	59	8
Third dose of diphtheria–tetanus–pertussis–Hib for children, % coverage	2008	86	85	5		2012	92	78	4
Tuberculosis treatment success rate, %	2008	88	87	2		2012	90	86	1
Antiretroviral coverage, %	NA	NA	NA	NA		2012	65	64	5

Hib: *Haemophilus influenzae* type b; NA: not available; PPP: parity purchasing power.

^a^ Not including Zanzibar.

^b^ Burundi, Democratic Republic of the Congo, Ethiopia, Kenya, Malawi, Mozambique, Rwanda, Uganda, Zambia and Zimbabwe.

^c^ United Republic of Tanzania’s position among the total 11 countries.

^d^ The relevant data were only available for eight of the 11 countries included in the comparison.

Data sources: World Health Organization,^18^ the Global Health Observatory database^22^ and Demographic and Health Surveys.^23^

## Discussion

Overall, the data collected on the progress made during the first half of the implementation of the United Republic of Tanzania’s strategic plan for health in 2009–2015 are encouraging. During the period, major progress was made in child mortality and nutrition and the coverage of interventions to improve child health and control HIV, tuberculosis and malaria. Over the same period, however, there were only minor improvements in maternal and neonatal health and these did not match those in several other countries in eastern or central Africa. The findings of our review resulted in a set of revised recommendations for the second half of the plan’s implementation, such as the need for greater emphasis on maternal and neonatal health services.

For any national plan that is to be implemented over several years, accountability and implementation need to be guided by up-to-date data on the relevant long- and short-term trends. Our analysis illustrates how a range of data sources can be used to obtain a comprehensive picture of the progress being made towards the targets of such a plan. Recent population-based health surveys were used to assess trends in several indicators such as mortality, intervention coverage and health behaviours – according to equity stratifiers – and to verify statistics derived from studies based in health facilities. Health facility data played a major role in our analysis because they were available for the year preceding the midterm review, allowed considerable geographical disaggregation and included all of the relevant information available on tuberculosis and HIV treatment. In addition, recent surveys of health facilities allowed us to assess service readiness and, therefore, the performance of the service delivery system.^14^ Reliable administrative data were needed to assess trends in the financial and health workforce indicators.

Comparisons with similar countries can provide a useful perspective on the performance of a particular country’s health system. In our choice comparison countries, we aimed to avoid an excessive preoccupation with country rankings.^23^ Gaps in the data meant that we had to rely on the predictions of WHO or another United Nations agency for the between-country comparisons of several financial and health status indicators. Although the United Republic of Tanzania is relatively data-rich, we struggled to find enough data – or, at least, enough data of adequate quality – on several topics of interest. Such gaps are likely to be more prominent in countries with less frequent national health surveys or with poorly functioning routine health management information systems. The quality of the data collected routinely from health facilities is often very variable and such data need to be assessed carefully. A change in the system used to collect facility data, from a paper-based system to a web-based system,^24^ should facilitate the systematic management, quality assessment and analysis of facility data.

A system of national health accounts and a comprehensive system for the registration of health workers are both necessary for the implementation of any national health plan. Although the indicators and targets that we investigated may not have been sufficient to provide a comprehensive picture of progress and performance, we made no attempt to gather data on topics that were beyond the scope of the plan. Some of these areas, such as noncommunicable diseases, may nonetheless have major and growing impacts on the health of Tanzanians.^25^

In any midterm analysis, inequalities as the result of demographic, geographical or socioeconomic characteristics should be considered. A lack of district-level data of adequate quality meant that we were obliged to focus on regional differences in our analysis of geographical inequalities,^19^ even though analysis at district level – i.e. the level at which United Republic of Tanzanian resources for health are allocated – may have been preferable. The regional data we used came from large samples and from population surveys and health facilities. Although this is not the case in the United Republic of Tanzania, the districts of some countries, such as South Africa, have such large populations that district-level analysis of data from health facilities is both feasible and useful.^26^

This analysis should help the United Republic of Tanzania develop a single strong monitoring and evaluation platform for its national strategic plans and major health programmes.^7^ Such a platform, if adequately funded, could collect and disseminate useful data of good quality and transparency and form the basis for all global reporting to donors.
